# Efficient biosynthesis of pinosylvin from lignin-derived cinnamic acid by metabolic engineering of *Escherichia coli*

**DOI:** 10.1186/s13068-022-02236-5

**Published:** 2022-12-12

**Authors:** Yueli Hu, Chen Zhang, Lihua Zou, Zhaojuan Zheng, Jia Ouyang

**Affiliations:** 1 Jiangsu Co-Innovation Centre of Efficient Processing and Utilization of Forest Resources, Nanjing Forestry University, Nanjing, 210037 People’s Republic of China; 2 College of Chemical Engineering, Nanjing Forestry University, Nanjing, 210037 People’s Republic of China

**Keywords:** Pinosylvin, Stilbene synthase, 4-Coumarate-CoA ligase, *Trans*-cinnamic acid, Biosynthesis

## Abstract

**Background:**

The conversion of lignin-derived aromatic monomers into valuable chemicals has promising potential to improve the economic competitiveness of biomass biorefineries. Pinosylvin is an attractive pharmaceutical with multiple promising biological activities.

**Results:**

Herein, *Escherichia coli* was engineered to convert the lignin-derived standard model monomer cinnamic acid into pinosylvin by introducing two novel enzymes from the wood plant: stilbene synthase from *Pinus pinea* (PpSTS) and 4-Coumarate-CoA ligase from *Populus trichocarpa* (Ptr4CL4)*.* The expression of Ptr4CL4 drastically improved the production of pinosylvin (42.5 ± 1.1 mg/L), achieving values 15.7-fold higher than that of Ptr4CL5 (another 4-Coumarate-CoA ligase from *Populus trichocarpa*) in the absence of cerulenin. By adjusting the expression strategy, the optimized engineered strain produced pinosylvin at 153.7 ± 2.2 mg/L with an extremely high yield of 1.20 ± 0.02 mg/mg cinnamic acid in the presence of cerulenin, which is 83.9% ± 1.17 of the theoretical yield. This is the highest reported pinosylvin yield directly from cinnamic acid to date.

**Conclusion:**

Our work highlights the feasibility of microbial production of pinosylvin from cinnamic acid and paves the way for converting lignin-related aromatics to valuable chemicals.

**Supplementary Information:**

The online version contains supplementary material available at 10.1186/s13068-022-02236-5.

## Background

As the lignocellulose volume is the largest renewable resource on earth, its biorefinery has attracted extensive attention for its sustainability [1]. Amongst the three components of lignocellulose, its carbohydrate contents (cellulose and hemicellulose) could be used for biofuel, biochemical and biomaterial production. However, lignin, accounting for 15–40%, is still difficult to degrade and utilize [2, 3]. This greatly limits the economic competitiveness of biomass biorefineries. Exploring lignin biorefineries for aromatic chemical production has promising potential to improve the lignin economic competitiveness of biomass biorefineries. According to the previous studies, many aromatic monomers from lignin can be efficiently released by pretreatment and thermochemical depolymerization [4]. These compounds mainly include various phenolic acids, such as ferulic acid, *p*-coumaric acid and cinnamic acid. Thus, converting these accessible lignin-related phenolic acids to value-added products contributes to realizing the value-added utilization of lignin waste. Our findings open up possibilities for the practical biosynthesis of natural pinosylvin from lignin-derived standard model monomer cinnamic acid at industrial scale.

Pinosylvin (*trans*-3,5-dihydroxystilbene) is an important stilbene compound that is mostly present in the heartwood of coniferous trees. Its natural function mainly protects plants against microbial and fungal decay [5]. Recent studies have shown that it has multiple promising biological activities, including anticancer, anti-cardiovascular, antioxidation, anti-inflammatory and antibacterial abilities [6–9]. Pinosylvin can be found in plant tissues, pine leaves and fruits (*Pinus densiflora*) [10–12]. However, several shortcomings have increased its economic and labour costs for market demand, including a low concentration (1–40 mg/g pine wood), seasonal and regional variations and the existence of structural analogues [13, 14]. In addition, from an industrial perspective, large-scale plant extraction would lead to a decrease in the vegetation coverage and damage to the ecological environment, which is contrary to the goal of sustainable development [15]. Hence, constructing engineered cells by synthetic biology and metabolic engineering technologies is desired and has been demonstrated to be a more environmentally friendly and cost-efficient platform for pinosylvin production.

**Fig. 1:**
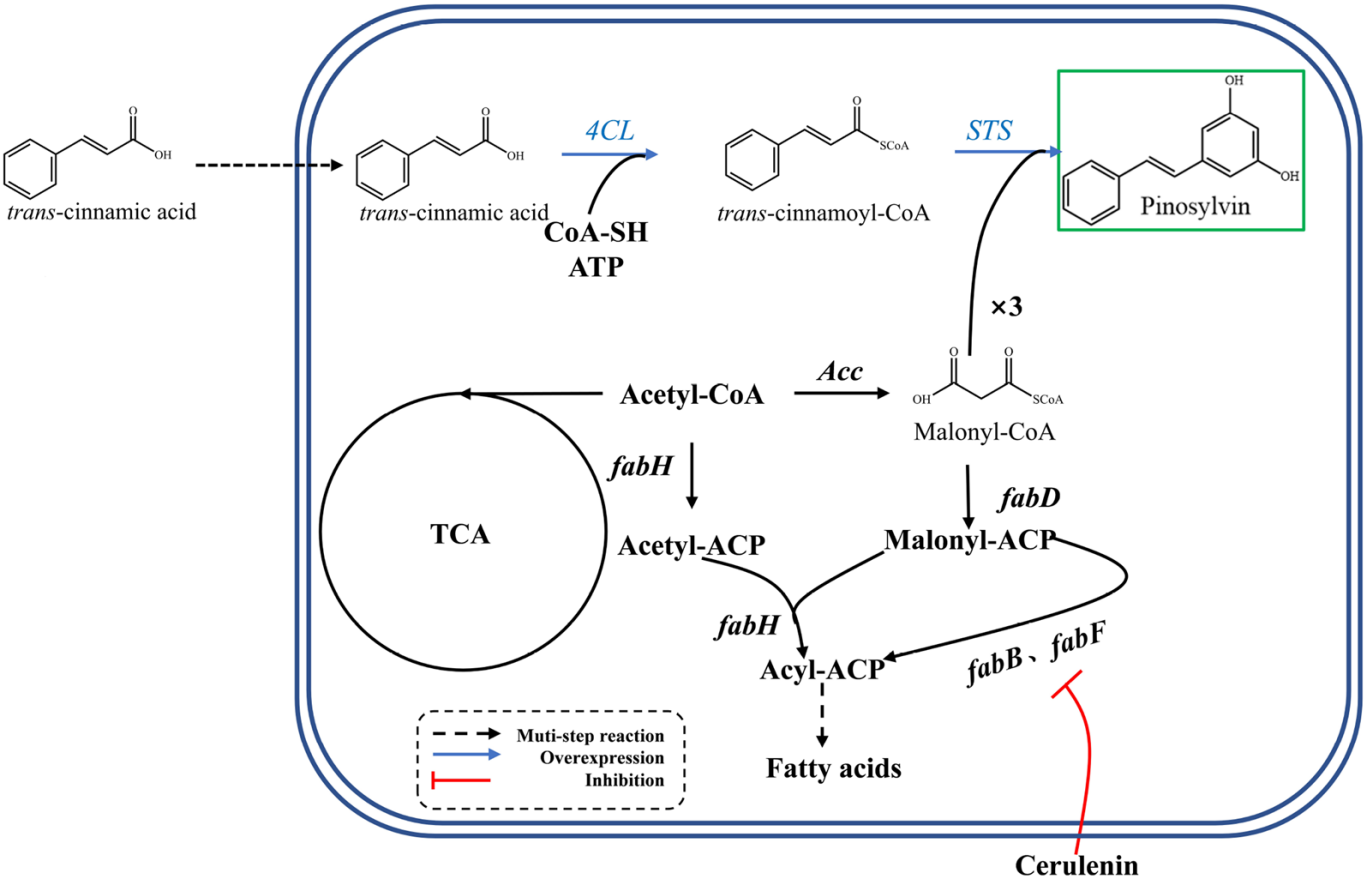
Pinosylvin biosynthetic pathway and malonyl-CoA metabolic networks. *4CL*, encoding 4-coumarate-CoA ligase; *STS*, encoding stilbene synthase; *Acc*, encoding acetyl-CoA carboxylase; *fabD*, encoding malonyl-CoA: ACP transacylase; *fabB*, encoding β-ketoacyl-ACP synthase I; *fabF*, encoding β-ketoacyl-ACP synthase II; *fabH*, encoding β-ketoacyl-ACP synthase III. The blue arrows and the red lines represent overexpression and inhibition of genes or proteins. The dotted lines indicate multistep reaction

The basic skeleton of pinosylvin is composed of a B ring from a *trans*-cinnamoyl coenzyme A (*trans*-cinnamoyl-CoA) and an A ring formed by the cyclization of three malonyl coenzyme A (malonyl-CoA) molecules. Phenylalanine ammonia lyase (PAL; EC 4.3.1.24) converts l-phenylalanine synthesized by the shikimate pathway to *trans*-cinnamic acid. *Trans*-cinnamic acid is CoA-activated by a 4-coumarate-CoA ligase (4CL; EC 6.2.1.12), yielding *trans*-cinnamoyl-CoA. Subsequently, pinosylvin is specifically biosynthesized by stilbene synthase (STS; EC 2.3.1.146), catalysing the condensation of three malonyl-CoA molecules with *trans*-cinnamoyl-CoA (Fig. 1) [12]. In plants, pinosylvin is converted from *trans*-cinnamic acid [6, 16], a class of natural plant intermediates from the lignin metabolism pathway [17, 18]. 4CL and STS are involved in the biosynthetic pathway of pinosylvin from *trans*-cinnamic acid. To date, according to the plant pathway, the production of pinosylvin using engineered microorganisms has been studied [19, 20]. The supply of malonyl-CoA has been found to be critical for pinosylvin production [21, 22]. Improving the level of intracellular malonyl-CoA had a remarkable positive effect on the production of pinosylvin [23]. However, although a long and complex artificial pathway of pinosylvin production from glucose was constructed and optimized, the product titre and yield were still not satisfactory. Assembling the full pathway from glucose to pinosylvin resulted in a yield of only 56.2 mg/g glucose even through rational modular design of the metabolic network [24]. When the substrate was replaced by *trans*-cinnamic acid, the maximum yield of pinosylvin reached 0.64 mg/mg *trans*-cinnamic acid [19]. Therefore, improving the yield is of great interest for pinosylvin production.

The aim of this work was to engineer *E. coli* for the production of pinosylvin from *trans*-cinnamic acid with both high titre and high yield. To achieve this, different STSs from *Pinus pinea* and 4CLs from *Populus trichocarpa* were screened, and the most suitable combination was identified by comparing the production of pinosylvin. Based on the results, the mechanism by which the enzyme 4CL regulates the pathway was explored. Thereafter, the metabolic engineering pathway was optimized by genetic manipulation. Finally, the culture conditions for pinosylvin production in engineered *E. coli* were optimized. This work thus demonstrated highly efficient pinosylvin production from cinnamic acid, with the highest yield to date. The yield was 1.20 ± 0.02 mg/mg *trans*-cinnamic acid, corresponding to 83.9% ± 1.17 of the theoretical yield.

## Results and discussion

### Genomic mining of stilbene synthases from *Pinus* for pinosylvin production

**Fig. 2:**
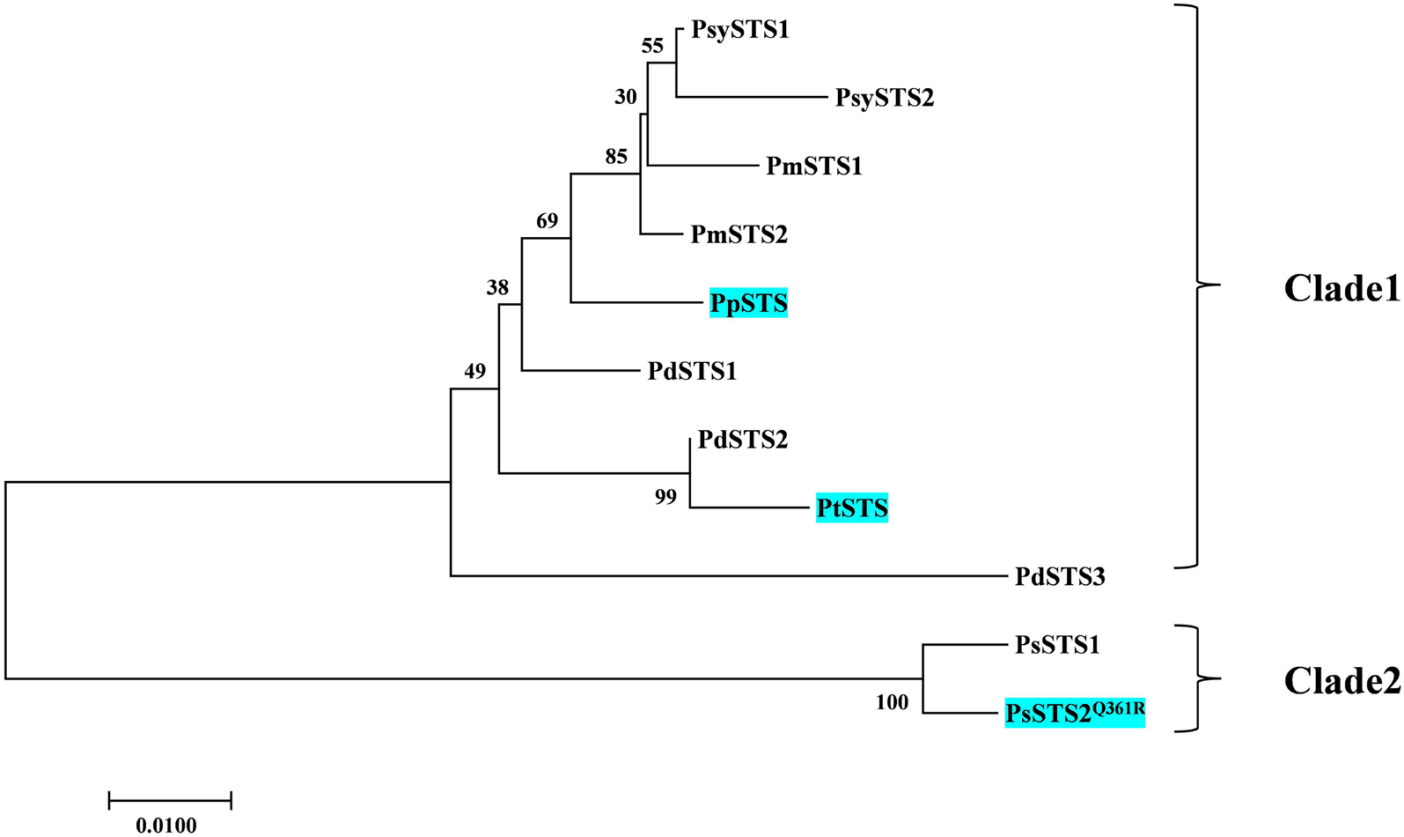
Stilbene synthase sequence analysis. Phylogenetic tree constructed for the selected STS sequences from various pines based on the neighbour-joining method. All STSs used were as follows: PsySTS, STS from *Pinus sylvestris*; PmSTS, STS from *Pinus massoniana*; PpSTS, STS from *Pinus pinea*; PdSTS, STS from *Pinus densiflora*; PtSTS, STS from *Pinus thunbergii*; PsSTS, STS from *Pinus strobus*. The accession numbers of the proteins are listed in Additional file 1: Table S1

According to the biosynthetic pathway of pinosylvin in plants, both 4CL and STS are necessary for pinosylvin production from *trans*-cinnamic acid. Previous studies suggested that the biosynthesis of pinosylvin from *trans*-cinnamoyl-CoA, which is in the charge of STS, is the limiting step [25, 26]. Currently, many STSs have been characterized from plants, and most of them have been applied in the formation of resveratrol [27, 28]. However, only AhSTS (STS from *Arachis hypogaea*), VvSTS (STS from *Vitis vinifera*) and PsSTS (STS from *Pinus strobus*) were successfully expressed in *E. coli* for pinosylvin production [12, 24, 29, 30]. Pinosylvin is a stilbene that can be predominantly found in the heartwood of coniferous trees of the genus *Pinus*. To mine more STSs for use, 11 STS genes from six *Pinus* species were screened from NCBI in this study (Additional file 1: Table S1). Amongst these proteins, PsySTS1, PmSTS, PdSTS and PsSTS2 have been reported to display catalytic activities for *trans*-cinnamoyl-CoA [15, 16, 27, 31]. As the phylogenetic tree showed (Fig. 2), all these STSs were grouped into two clusters. PsSTS1 and PsSTS2^Q361R^ from *Pinus strobus* shared the same clade. PsSTS2 was confirmed to have good activity towards *trans*-cinnamoyl-CoA, and its single mutant PsSTS2^Q361R^ displayed higher activity towards *trans*-cinnamoyl-CoA, which would increase pinosylvin production [15, 16]. Meanwhile, PpSTS was not clearly identified in a previous study. However, it demonstrated high protein identity (> 98%) with PsySTS1, PmSTS1-2 and PdSTS1*,* which indicated that it might be responsible for the conversion of *trans*-cinnamoyl-CoA to pinosylvin. In addition, PtSTS shares a small clade with PdSTS2 which has the ability to convert *trans*-cinnamoyl-CoA to pinosylvin [31]. For the above reasons, we selected PsSTS2^Q361R^, PpSTS and PtSTS as candidates for pinosylvin synthases for further study.

**Fig. 3:**
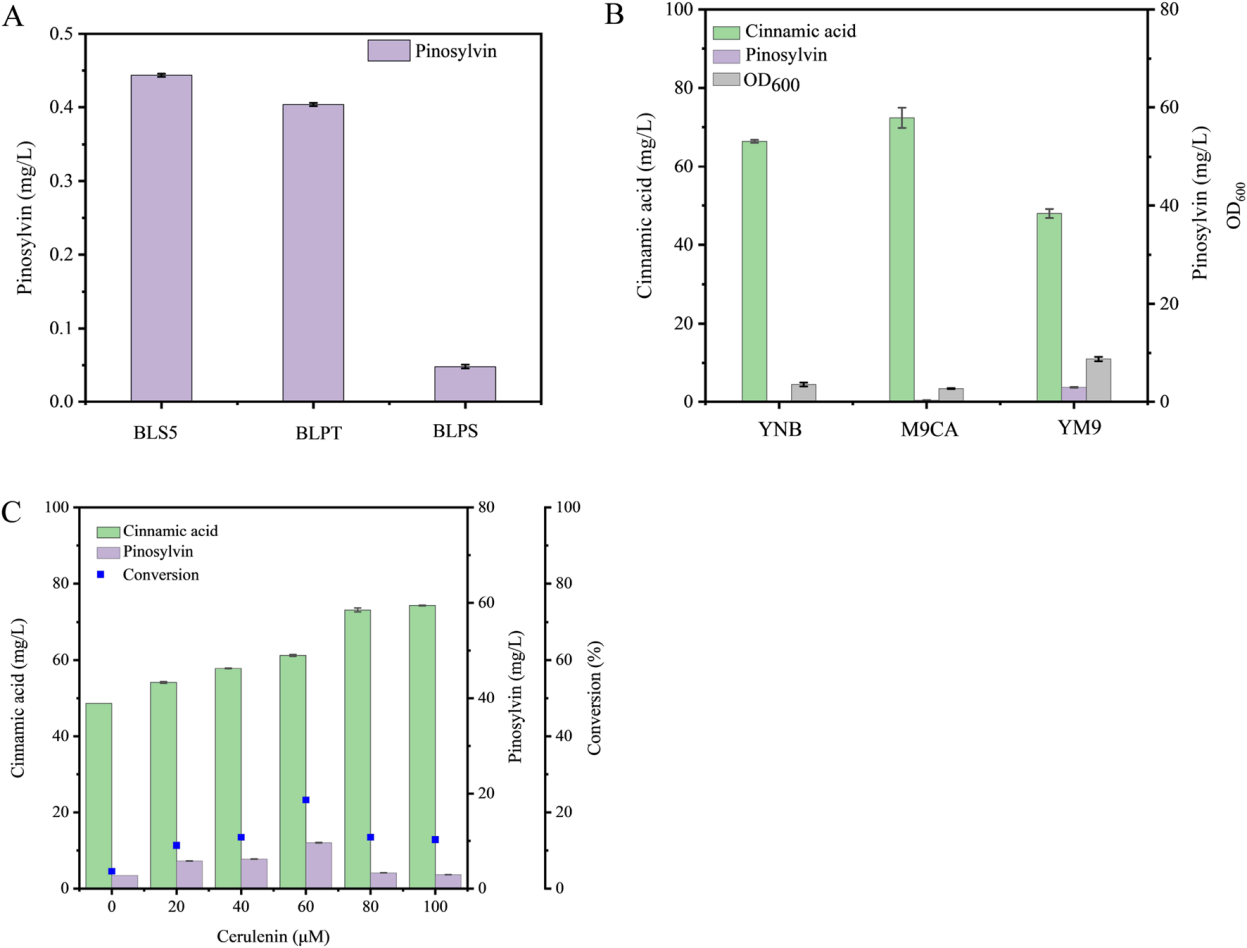
For comparison of recombinant *E. coli* metabolites, 90 mg/L *trans*-cinnamic acid was supplied for pinosylvin production. **A** Comparison of pinosylvin produced by different recombinant *E. coli* BLPS, BLPT and BLS5 in M9CA medium. **B** Pinosylvin production, remaining *trans*-cinnamic acid and OD_600_ of *E. coli* BLS5 cultivated in different media for 48 h. **C** Pinosylvin production and remaining *trans*-cinnamic acid of *E. coli* BLS5 under different concentrations of cerulenin in YM9 medium for 48 h

To assess the performance of the three candidate enzymes, three engineered strains, BLS5 (BL21(DE3) harbouring genes *PpSTS* and *Ptr4CL5*), BLPT (BL21 (DE3) harbouring genes *PtSTS* and *Ptr4CL5*) and BLPS (BL21 (DE3) harbouring genes *PsSTS*^*Q361R*^ and *Ptr4CL5*), were constructed for pinosylvin production (Table 1). Therein, Ptr4CL5 was used for converting *trans*-cinnamic acid to *trans*-cinnamoyl-CoA, which displayed a high activity towards *trans*-cinnamic acid in our previous report [32]. Three strains were cultivated in M9CA medium using *trans*-cinnamic acid as substrates. As shown in Fig. 3A, all of them showed a certain capacity for pinosylvin production, which confirmed that Ptr4CL5 was indeed active to *trans*-cinnamic acid. As control, BL21(DE3) with plasmid pRSFDuet-1 did not synthesize pinosylvin. By comparison, BLS5 demonstrated the highest ability to synthesize pinosylvin, obtaining 0.44 ± 0.03 mg/L pinosylvin. Therefore, PpSTS was considered to be the best for pinosylvin production. Thereafter, we speculated that the M9CA medium (which uses glucose as a carbon source) did not provide enough nutrients for growth, which led to the trace amount of pinosylvin. Thus, YNB (which uses glucose as a carbon source) and YM9 (which uses glycerol as a carbon source and yeast extract was added) media were used (Fig. 3B). When YM9 medium was used, along with the improved growth, the concentration of product increased to 2.98 ± 0.08 mg/L, which was 571% ± 0.16 higher than that achieved with M9CA. Meanwhile, BLS5 cultivated in YM9 consumed the most *trans*-cinnamic acid. Nevertheless, only 46.1% of *trans*-cinnamic acid was consumed in YM9 medium, and the yield was obviously unsatisfactory. It was considered that the low conversion may be due to an insufficient supply of malonyl-CoA. Malonyl-CoA was reported to be a key precursor for preparing stilbene and flavone, including pinosylvin [20]. As illustrated in Fig. 1, decreasing malonyl-CoA involved in the fatty acid synthesis pathway would be an effective method for malonyl-CoA supply [33]. Owing to the inhibitory effect of cerulenin on fatty acid synthesis [34, 35], cerulenin was added to increase the intracellular level of malonyl-CoA. As expected, pinosylvin reached 9.61 ± 0.06 mg/L, which increased by 260% ± 0.45 in the presence of 60 μM cerulenin (Fig. 3C). However, the inhibition of fatty acid synthesis resulted in the poor growth of BLS5 (Additional file 1: Fig. S1), which might influence the total expression amounts of proteins in *E. coli*. Hence, even if cerulenin addition enhanced the supplementation of precursor malonyl-CoA, the low yield still suggested that exploring other 4CLs might be more beneficial for pinosylvin production.

**Table 1:** Strains and plasmids used in this study

	Characteristics	Source
*Strains*		
*E. coli* BL21 (DE3)	F^−^ ompT hsdSB (rB^−^ mB^−^) gal (λ cI857 ind1 sam7 nin5 lacI lacUV5-T7 gene1), dcm (DE3)	Transgen
*E. coli* Trans1-T1	F^−^ ϕ80 (lacZ) ΔM15 ΔlacX 74 hsdR(rk^−^mk^−^) ΔrecA 1398endA1tonA	Transgen
*E. coli* BL4	BL21(DE3) carrying pE-4CL4	Laboratory
*E. coli* BL5	BL21(DE3) carrying pE-4CL5	Laboratory
*E. coli* BLS5	BL21(DE3) carrying pR-PpSTS-Ptr4CL5	This study
*E. coli* BLPT	BL21(DE3) carrying pR-PtSTS-Ptr4CL5	This study
*E. coli* BLPS	BL21(DE3) carrying pR-PsSTS2^Q361R^-Ptr4CL5	This study
*E. coli* BR4S	BL21(DE3) carrying pR-Ptr4CL4-PpSTS	This study
*E. coli* BRS4	BL21(DE3) carrying pR-PpSTS-Ptr4CL4	This study
*E. coli* BE4S	BL21(DE3) carrying pE-Ptr4CL4-PpSTS	This study
*E. coli* BES4	BL21(DE3) carrying pE-PpSTS-Ptr4CL4	This study
*E. coli* BC4S	BL21(DE3) carrying pC-Ptr4CL4-PpSTS	This study
*E. coli* BCS4	BL21(DE3) carrying pC-PpSTS-Ptr4CL4	This study
*E. coli* BRT4S	BL21(DE3) carrying pRT-Ptr4CL4-PpSTS	This study
*E. coli* BRTS4	BL21(DE3) carrying pRT-PpSTS-Ptr4CL4	This study
*E. coli* BET4S	BL21(DE3) carrying pET-Ptr4CL4-PpSTS	This study
*E. coli* BETS4	BL21(DE3) carrying pET-PpSTS-Ptr4CL4	This study
*E. coli* BCT4S	BL21(DE3) carrying pCT-Ptr4CL4-PpSTS	This study
*E. coli* BCTS4	BL21(DE3) carrying pCT-PpSTS-Ptr4CL4	This study
*Plasmids*		
pRSFDuet-1	RSF ori with P_T7_; Kan^r^	Novagen
pCDFDuet-1	CDF ori with P_T7_; Sm^r^	Novagen
pETDuet-1	PBR322 ori with P_T7_; Amp^r^	Novagen
pE-4CL4	pETDuet-1 with *Ptr4CL4*	Laboratory
pE-4CL5	pETDuet-1 with *Ptr4CL5*	Laboratory
pR-PpSTS	pRSFDuet-1 with *PpSTS*	Generay Biotech
pR-PtSTS	pRSFDuet-1 with *PtSTS*	Generay Biotech
pR-PsSTS2^Q361R^	pRSFDuet-1 with *PsSTS2*^*Q361R*^	Generay Biotech
pR-PpSTS-Ptr4CL5	pRSFDuet-1 with P_T7_-*PpSTS* and P_T7_-*Ptr4CL5*	This study
pR-PtSTS-Ptr4CL5	pRSFDuet-1 with P_T7_-*PtSTS* and P_T7_-*Ptr4CL5*	This study
pR-PsSTS2^Q361R^-Ptr4CL5	pRSFDuet-1 with P_T7_-*PsSTS2*^*Q361R*^ and P_T7_-*Ptr4CL5*	This study
pR-PpSTS-Ptr4CL4	pRSFDuet-1 with P_T7_-*PpSTS* and P_T7_-*Ptr4CL4*	This study
pR-Ptr4CL4-PpSTS	pRSFDuet-1 with P_T7_-*Ptr4CL4* and P_T7_-*PpSTS*	This study
pE-PpSTS-Ptr4CL4	pETDuet-1 with P_T7_-*PpSTS* and P_T7_-*Ptr4CL4*	This study
pE-Ptr4CL4-PpSTS	pETDuet-1 with P_T7_-*Ptr4CL4* and P_T7_-*PpSTS*	This study
pC-PpSTS-Ptr4CL4	pCDFDuet-1 with P_T7_-*PpSTS* and P_T7_-*Ptr4CL4*	This study
pC-Ptr4CL4-PpSTS	pCDFDuet-1 with P_T7_-*Ptr4CL4* and P_T7_-*PpSTS*	This study
pRT-PpSTS-Ptr4CL4	pRSFDuet-1 with P_T7_-*PpSTS*-*Ptr4CL4*	This study
pRT-Ptr4CL4-PpSTS	pRSFDuet-1 with P_T7_ -*Ptr4CL4*-*PpSTS*	This study
pET-PpSTS-Ptr4CL4	pETDuet-1 with P_T7_-*PpSTS*-*Ptr4CL4*	This study
pET-Ptr4CL4-PpSTS	pETDuet-1 with P_T7_ -*Ptr4CL4*-*PpSTS*	This study
pCT-PpSTS-Ptr4CL4	pCDFDuet-1 with P_T7_-*PpSTS*-*Ptr4CL4*	This study
pCT-Ptr4CL4-PpSTS	pCDFDuet-1 with P_T7_-*Ptr4CL4*-*PpSTS*	This study

### Improving pinosylvin conversion from *trans*-cinnamic acid by altering 4-coumarate-CoA ligase

**Fig. 4:**
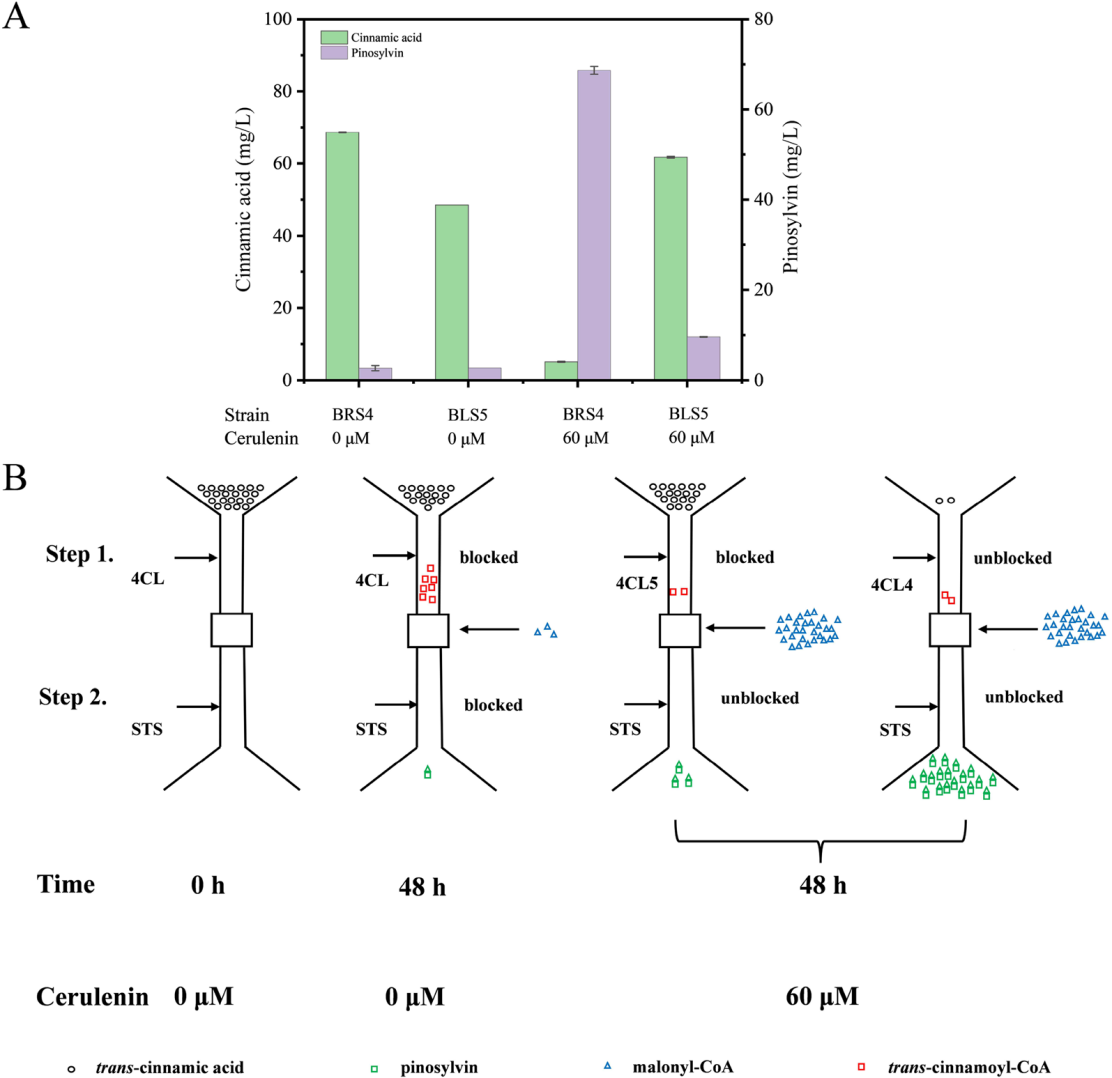
Effects of different 4CLs on pinosylvin production. **A** Remaining *trans*-cinnamic acid and pinosylvin produced by different *E. coli* strains BLS5 and BRS4 under 0 and 60 μM cerulenin. The strains were cultivated at 30 °C for 48 h in YM9 medium and 90 mg/L *trans*-cinnamic acid was added. **B** The assumed regulatory mechanism involved in the biosynthesis system of pinosylvin from *trans*-cinnamic acid by engineered *E. coli.* The black circles indicate *trans*-cinnamic acid, the red squares indicate *trans*-cinnamoyl-CoA, the blue triangles indicate malonyl-CoA, the green pentagons indicate pinosylvin

4CL is the main branch point enzyme that generates activated thioesters. Many plants have a large number of 4CLs, which connect phenylpropanoids to different product pathways. In our previous work, another 4CL from *Populus trichocarpa* (Ptr4CL4) was also found to be active to *trans*-cinnamic acid. Although it has lower activity towards *trans*-cinnamic acid, its primary function is confirmed to channel-activated 4-coumarate to different branch pathways of flavonoid synthesis, and its regulation is more complicated than that of Ptr4CL5 [32]. Thus, it was considered to be introduced into the pinosylvin biopathway by substituting for Ptr4CL5. As shown in Fig. 4A, *E. coli* BRS4 (harbouring Ptr4CL4 and PpSTS) produced 2.69 ± 0.58 mg/L pinosylvin, which did not bring an obvious change for pinosylvin production without cerulenin (*p > *0.05). Moreover, the low activity of Ptr4CL4 led to more *trans*-cinnamic acid accumulation. However, unexpectedly, cerulenin addition resulted in a remarkable difference between Ptr4CL4 and Ptr4CL5. For Ptr4CL5, cerulenin addition resulted in less *trans*-cinnamic acid consumption, which might be due to the decreasing OD_600_ and lower protein expression level (Additional file 1: Fig. S1). However, the combination of Ptr4CL4 and PpSTS achieved the highest consumption of *trans*-cinnamic acid in the presence of cerulenin, with only 5.13 ± 0.50 mg/L left after fermentation of 48 h. Meanwhile, the concentration of pinosylvin for *E. coli* BRS4 increased dramatically to 68.64 ± 0.87 mg/L, which was approximately seven times that of *E. coli* BLS5.

The above results indicated that, in the absence of cerulenin, insufficient levels of malonyl-CoA block pinosylvin biosynthesis in the 2nd step [36–38], which might result a certain amount of *trans*-cinnamoyl-CoA accumulation (Fig. 4B). The addition of cerulenin had a positive effect on the supplementation of malonyl-CoA [39]. When 60 μM cerulenin was added, the 2nd step catalysis by STS was unblocked, and an increase in pinosylvin production was observed. The improved production of pinosylvin using Ptr4CL5 seemed to be only from the accumulated *trans*-cinnamoyl-CoA, because no more *trans*-cinnamic acid was consumed (Fig. 4A). In the presence of cerulenin, the combination of Ptr4CL4 and STS was more suitable. Once cerulenin was added, the overall biopathway was unobstructed, and the yield of pinosylvin increased from 0.03 mg/mg *trans*-cinnamic acid to 0.76 mg/mg *trans*-cinnamic acid.

According to the previous studies, most 4CLs do not effectively convert *trans*-cinnamic acid into the corresponding ester in vitro [15, 32]. Only a few 4CLs, including Sc4CL, At4CL1, Pc4CL and Ptr4CL5, were used for the biosynthesis of *trans*-cinnamic acid derivatives [40]. However, their results were poor [41]. Our results showed that the wood-derived gene *Ptr4CL4* is more efficient than the other genes for pinosylvin production. This finding underlines the importance of altering variants of 4CL in the pathway of pinosylvin biosynthesis. In the subsequent experiment, a combination of Ptr4CL4 and PpSTS was used for pinosylvin production.

### Optimization of the pinosylvin-producing strain by combinatorial engineering

**Fig. 5:**
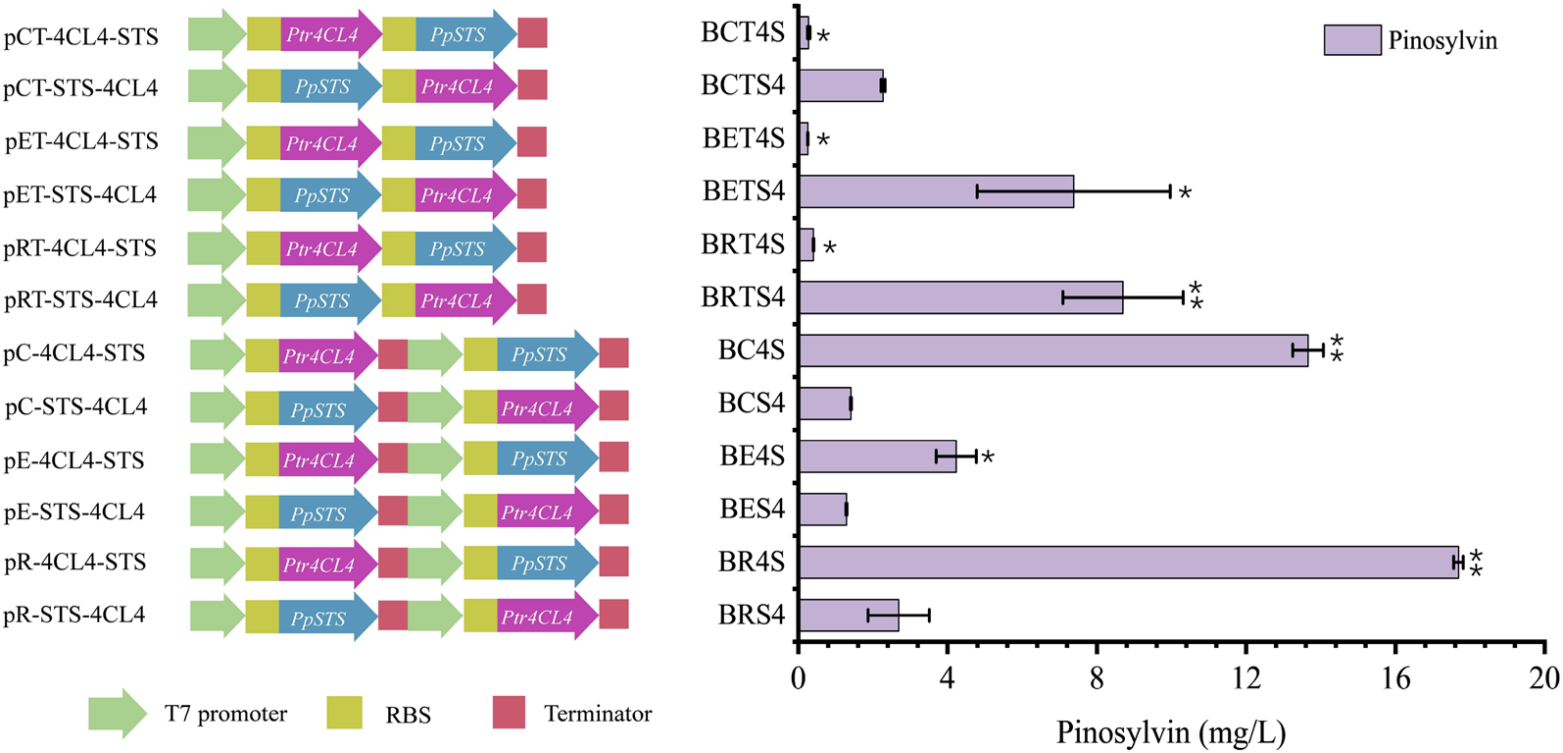
Pinosylvin production by recombinant *E. coli* harbouring different modules. The strains were cultivated at 30 °C for 48 h in YM9 medium, and 90 mg/L *trans*-cinnamic acid was added. The data represent the means of two replicates, and error bars represent standard deviations. “*” indicates *p* < 0.05, “**” indicates *p* < 0.01

Generally, basic modular optimization, such as promoter, gene copy number and gene order, is significant in reconstructing an effective metabolic pathway in microbes [42]. Thus, our strain was further engineered to balance the catalytic activities of Ptr4CL4 and PpSTS. A series of strains harbouring Ptr4CL4 and PpSTS were constructed by adjusting the expression strategy (Table 1). As shown in Fig. 5, three different plasmids, pCDFDuet-1 (CDF origin), pETDuet-1 (pBR322 origin), and pRSFDuet-1 (RSF origin), were used to regulate module expression, corresponding to gene copy numbers of 20, 40 and 100, respectively, according to previous reports [24]. When only one T7 promoter was employed, strains harbouring the *PpSTS* gene in front of the Ptr4CL4 gene produced more pinosylvin than those in the opposite order. This meant that the STS close to the T7 promoter was suitable for the pinosylvin pathway. Pinosylvin biosynthesis could be hampered by low heterologous pathway activity [43]. With the same T7 promoter, the production with a high-copy-number plasmid was approximately threefold greater than that with a low-copy-number plasmid (comparison between strains BRTS4 and BCTS4). However, when two T7 promoters were employed in strains, *Ptr4CL4* placed in front of *PpSTS* tended to produce more pinosylvin. Comparing the protein expression levels of the two strains BR4S and BRS4, we found that the solubility of Ptr4CL4 and PpSTS in BRS4 was much lower than that in BR4S (Additional file 1: Fig. S2), which partly resulted in an approximately sixfold change in production. It was concluded that both the order of the two genes and the copy number of the vector play important roles in protein functional folding and expression levels, which may lead to low pinosylvin synthesis. Similar to this phenomenon, Zhou et al. produced the fusion proteins ERG20-BTS1 and BTS1-ERG20 and found that BTS1-ERG20 resulted in increased miltiradiene production compared with ERG20-BTS1 [44]. Overall, *E. coli* BR4S produced 17.69 ± 0.13 mg/L pinosylvin from 90 mg/L *trans*-cinnamic acid in the absence of cerulenin, which was the highest yield amongst these 12 constructed strains.

### Efficient pinosylvin production by strain BR4S

**Fig. 6:**
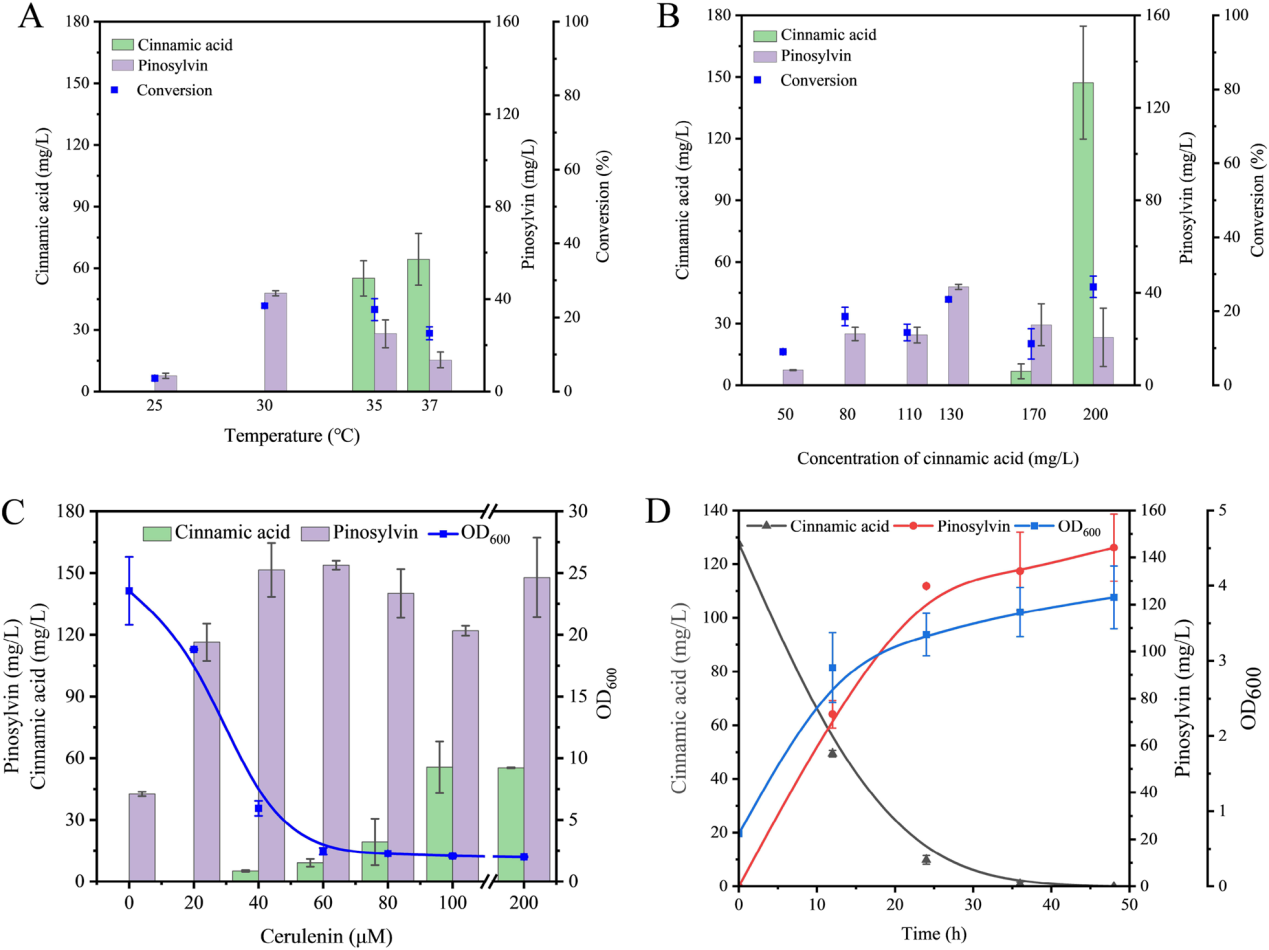
Bioconversion of *trans*-cinnamic acid to pinosylvin with *E. coli* BR4S. **A** Effect of temperature on the synthesis of pinosylvin. *Trans*-cinnamic acid concentration was 130 mg/L and no cerulenin was added. **B** Effect of *trans*-cinnamic acid on the synthesis of pinosylvin. The temperature was 30 °C and no cerulenin was added. **C** Effect of cerulenin on the synthesis of pinosylvin. The temperature was 30 °C and the concentration of *trans*-cinnamic acid was same as (**A**). **D** The course of BR4S with 60 μM cerulenin

Finally, we investigated the effect of fermentation conditions on pinosylvin production efficiency, such as temperature, *trans*-cinnamic acid dosage and cerulenin concentration. As shown in Fig. 6A, pinosylvin production by BR4S reached the highest titre of 42.5 ± 1.1 mg/L at 30 °C. In contrast to the performance of BRS4 in Fig. 4A, the complete consumption of *trans*-cinnamic acid at 25 °C and 30 °C indicated that the expression of 4CL in BR4S increased greatly and that the 2nd step became the bottleneck for production by optimizing the expression strategy. As shown in Fig. 6B, when using 50–130 mg/L *trans*-cinnamic acid initially, no residual *trans*-cinnamic acid was observed after 48 h of fermentation, suggesting that the activity of Ptr4CL4 is adequate to convert *trans*-cinnamic acid into its thioester. The pinosylvin concentration reached a maximum of 42.5 ± 1.1 mg/L at 130 mg/L *trans*-cinnamic acid. However, more *trans*-cinnamic acid seemed to trigger cell growth inhibition, which caused less *trans*-cinnamic acid to be consumed (Additional file 1: Fig. S3). Especially at 200 mg/L, a large amount of *trans*-cinnamic acid remained after fermentation. Finally, to analyse the contribution of cerulenin to production, cultivations were performed in which cerulenin (up to 200 μM) was supplemented. Adding 20–60 μM cerulenin greatly improved pinosylvin production but also influenced cell growth and protein expression due to its toxicity [45]. When cerulenin was added up to 40 μM, the accumulation of *trans*-cinnamic acid was observed. The highest pinosylvin production reached 153.7 ± 2.2 mg/L at 60 μM cerulenin (Fig. 6C). Meanwhile, the yield was 1.20 ± 0.02 mg/mg *trans*-cinnamic acid, corresponding to 83.9% ± 1.17 of the theoretical yield. The course of BR4S with 60 μM cerulenin is shown in Fig. 6D. A total of 130 mg/L *trans*-cinnamic acid was consumed within 36 h, whilst the highest pinosylvin production was observed at 48 h. It is worth noting that the growth of strains was seriously inhibited in the presence of cerulenin along with the enhancement of malonyl-CoA supply. At this time, the OD_600_ of the strains decreased to 28.0% of the original value, and pinosylvin production increased to three times the original value. As shown in Table 2, tens of mg/L pinosylvin was produced by different optimization tools. When compared with these studies, our study resulted in a higher titre and yield of pinosylvin from *trans*-cinnamic acid in the presence of cerulenin.

**Table 2:** Pinosylvin production from cinnamic acid in engineering bacteria

Host cell	Substrate concentration	Fermentation	Genetic modification	Product titre (mg/L)	Yield (mg/mg)	References
*E. coli*	130 mg/L	+ cerulenin	*Ptr4CL4*↑/*PpSTS*↑	153	1.201	This study
*Corynebacterium glutamicum*	740.9 mg/L	+ cerulenin	*AhSTS*↑/*Pc4CL*↑	121	0.163	[17]
*E. coli*	74.1 mg/L	−	*At4CL*↑/*VvSTS*↑/*fabD*↓	47	0.674	[19]
*E. coli*	444.5 mg/L	−	*Sc4CL*↑/*VvSTS*↑/*fabI*↓	52	0.118	[12]

## Conclusions

For the first time, the production of pinosylvin in a recombinant *E. coli* strain has been shown using a novel stilbene synthase PpSTS and a novel 4-coumarate-CoA ligase Ptr4CL4 from the wood plant. The choice of Ptr4CL4 is the key to increasing the yield of pinosylvin in the presence of cerulenin, which greatly benefits the biosynthesis of pinosylvin. By optimizing the expression strategy and culture conditions, we reached the highest pinosylvin production titre of 153.7 ± 2.2 mg/L at 60 μM cerulenin. To the best of our knowledge, the yield of 1.20 ± 0.02 mg/mg *trans*-cinnamic acid in the present study is the highest yield reported to date. Efficient biosynthesis of pinosylvin demonstrated potential application for the biosynthesis of products derived from cinnamic acid.

## Methods

### Strains, plasmids and media

All strains and plasmids used and constructed in this study are listed in Table 1. *E. coli* Trans-T1 was used as the general cloning host for plasmid construction and propagation. *E. coli* BL21(DE3) and its derivatives were used for enzyme expression and fermentation. The plasmids pRSFDuet-1, pETDuet-1 and pCDFDuet-1 were used as expression plasmids for recombinant plasmid construction.

All *E. coli* strains were grown with 90 mg/L *trans*-cinnamic acid in M9CA, YM9 or YNB medium for production. M9CA medium (1 L) consisted of 13.3 g M9CA broth (Sangon Biotech, Shanghai, China), 10 g glucose, 0.12 g MgSO_4_ and 0.5 mg VB1; YM9 medium (1 L) consisted of 11.3 g M9 salts (Sangon Biotech, Shanghai, China), 42 g MOPs (Sangon Biotech, Shanghai, China), 10 g yeast extract and 5% (v/v) glycerol [19]. For YNB medium, 100 mL of tenfold concentrated yeast nitrogen base without amino acids (Aladdin, Shanghai, China) and 10 g glucose was added to 900 mL base medium (6 g K_2_HPO_4_, 3 g KH_2_PO_4_, 10 g MOPS, pH 7.0).

### Sequence analysis

All protein coding sequences were obtained from the National Center for Biotechnology Information (NCBI) and BRENDA. The protein accession numbers used in this study are listed in Additional file 1: Table S1. Multiple sequence alignment was performed using Espript 3.0 (http://espript.ibcp.fr/ESPript/cgi-bin/ESPript.cgi). The phylogenetic tree was constructed by MEGA 7.0 software based on the neighbour-joining method.

### Pathway construction

The Ptr4CL4 (GenBank accession number EEF00197.1) and Ptr4CL5 (GenBank accession number EEE79804.2) sequences were codon optimized and synthesized by Generay Biotech. The gene products were excised via *Bam*HI and *Hind*III and then ligated into pETDuet-1 to yield pE-Ptr4CL5 and pE-Ptr4CL4, respectively.

PsSTS2^Q361R^ (the variant of PsSTS2 (GenBank accession number P48408.1), PtSTS (GenBank accession number AHK13302.1) and PpSTS (GenBank accession number ALN42233.1) sequences were codon optimized and synthesized by Generay Biotech. To construct the pinosylvin synthetic pathway, the high-copy-number vector pRSFDuet-1 was employed, generating plasmids pR-PsSTS2^Q361R^, pR-PtSTS and pR-PpSTS. Thereafter, *Ptr4CL5* was amplified by PCR using the primer pair 4CL5-f/4CL5-r and cloned into pR-PsSTS2^Q361R^, pR-PtSTS and pR-PpSTS through the *Nde*I and *Xho*I sites to generate plasmids pR-PsSTS2^Q361R^-Ptr4CL5, pR-PtSTS-Ptr4CL5 and pR-PpSTS-Ptr4CL5, respectively. In addition, the *Ptr4CL4* gene was amplified by PCR using the primer pair 4CL4-P-f/4CL4-P-r and cloned into pR-PpSTS through the *Nde*I and *Xho*I sites to generate the plasmid pR-PpSTS-Ptr4CL4.

To optimize modular expression, the *PpSTS* gene in plasmid pRSFDuet-1 was digested and cloned into plasmids pETDuet-1 and pCDFDuet-1 through the *Bam*HI and *Hind*III sites, yielding the intermediate expression plasmids pE-PpSTS and pC-PpSTS. The *Ptr4CL4* gene in plasmid pR-PpSTS-Ptr4CL4 was digested and cloned into plasmids pE-PpSTS and pC-PpSTS through the *Nde*I and *Xh*oI sites, yielding the expression plasmids pE-PpSTS-Ptr4CL4 and pC-PpSTS-Ptr4CL4. *Ptr4CL4* was digested from plasmid pE-Ptr4CL4 through *Bam*HI and *Hind*III and cloned into plasmids pRSFDuet-1 and pCDFDuet-1, generating plasmids pR-Ptr4CL4 and pC-Ptr4CL4, respectively. The *PpSTS* gene was amplified by PCR using the primer pair STS-f/STS-r and ligated with the linear DNAs *pE-Ptr4CL4*, *pR-Ptr4CL4* and *pC-Ptr4CL4,* which were amplified by PCR using the primer pair P-4-f/P-4-r, yielding the plasmids pE*-*Ptr4CL4-PpSTS, pR-Ptr4CL4-PpSTS and pC-Ptr4CL4-PpSTS, respectively. Plasmids pR-PpSTS-Ptr4CL4, pE-PpSTS and pC-PpSTS were amplified by PCR using primer pair plasmids-STS-f/plasmids-STS-r, yielding the linear DNAs *pR-PpSTS*, *pE-PpSTS* and *pC-PpSTS*. The *Ptr4CL4* gene was amplified by PCR using the primer pair RBS-4CL4-f/RBS-4CL4-r and ligated with the linear DNAs *pR-PpSTS*, *pE-PpSTS* and *pC-PpSTS*, generating plasmids pRT-PpSTS-Ptr4CL4, pET-PpSTS-Ptr4CL4 and pCT-PpSTS-Ptr4CL4. The *PpSTS* gene was amplified by PCR using the primer pair RBS-STS-f/RBS-STS-r, and the plasmids pR-Ptr4CL4, pE-Ptr4CL4 and pC-Ptr4CL4 were amplified by PCR using the primer pair plasmids-4CL4-f/plasmids-4CL4-r. *PpSTS* was ligated with the linear DNAs *pR-Ptr4CL4*, *pE-Ptr4CL4* and *pC-Ptr4CL4*, yielding plasmids pRT-Ptr4CL4-PpSTS, pET-Ptr4CL4-PpSTS and pCT-Ptr4CL4-PpSTS, respectively. All primers used in this study are listed in Additional file 1: Table S2.

### Microbial pinosylvin production

The recombinant strain was first cultivated in 50 mL fresh LB medium in 250 mL flasks for 12 h at 37 °C and 200 rpm. Then, these cells were diluted to an OD_600_ of 0.7 in 10 mL of M9CA, YM9 or YNB medium in 50 mL flasks at 200 rpm, and gene expression was induced with 1 mM IPTG. The cultivation was continued for 48 h at 30 °C, and 90 mg/L *trans*-cinnamic acid was added. After 48 h of fermentation, the fermentation broth was collected for analysis.

To test the effect of temperature, cultivation was performed with 1.0 mM IPTG and 130 mg/L *trans*-cinnamic acid. The effect of *trans*-cinnamic acid concentration was explored at 1.0 mM IPTG and 30 °C. To determine the effect of cerulenin on pinosylvin production, cultivation was carried out at 30 °C with 1.0 mM IPTG, 130 mg/L *trans*-cinnamic acid and different concentrations of cerulenin ranging from 0 to 100 μM. After 48 h of fermentation, the fermentation broth was collected for analysis.

For 48 h of optimized fermentation, samples were taken at 12, 24, 36 and 48 h intervals for subsequent analysis, and cultivation was carried out at 30 °C with 1.0 mM IPTG, 130 mg/L *trans*-cinnamic acid and 60 μM cerulenin. At least two duplicates were used for all experiments.

### Detection and quantification

Before HPLC analysis for compounds, the cell optical density (OD) of the culture was measured on a 752S spectrophotometer at 600 nm. Thereafter, the culture was centrifuged to remove the cell debris and further filtered through a 0.22 μm filter. The supernatant sample was used for *trans*-cinnamic acid analysis.

To analyse the amount of pinosylvin, 500 μL of supernatant was mixed with the same volume of ethyl acetate. The mixture was vortexed for 5 min and centrifuged at 13,400×*g* for 2 min. Then, 400 μL of the top organic layer was transferred to a new microtube and volatilized to dryness. Subsequently, the samples were resolubilized in 400 μL methanol for HPLC analysis.

HPLC analysis was carried out on an HPLC (Agilent 1260 Series, USA) using an Eclipse XDB-C18 column (250 mm × 4.6 mm, 5 μm). The UV‒Vis detector was set at 294 nm. The mobile phase consisted of 40% solvent A and 60% solvent B, where A was acetonitrile and B was water/acetic acid (98.5:1.5, v/v). The flow rate was set at 1.0 mL min^−1^. The column temperature was set to 30 ℃.

The calculation of the pinosylvin yield is shown below.

$${\text{Yield}}\,\left( {\text{mg/mg}} \right) = C_{{\text{p}}} /C_{{\text{t}}}$$

*C*_*p*_ is the concentration of pinosylvin in the medium; *C*_*t*_ is the initial concentration of *trans*-cinnamic acid. The unit of Cp and Ct is mg/L, and the theoretical yield was 1.43 mg/mg.

### Statistical analyses

All experiments were performed at least in two duplicates, and the results were expressed as means ± standard deviation (SD). The statistical analysis was performed using SPSS Statistics 20.0 (SPSS Inc., Chicago, IL, USA) with independent samples *t* test. Figures were drawn using OriginPro 2021(OriginLab, Northampton, MA, USA).

## Supplementary Information

**Additional file 1:** **Table S1.** GenBank IDs and sequences of stilbene synthases used in this study. **Table S2.** Primers used in this study. **Figure S1.** Growth curves of *E. coli* BLS5 cultured in YM9. **Figure S2.** SDS-PAGE of Ptr4CL4 and PpSTS in BRS4 and BR4S. **Figure S3.** The OD_600_ of BR4S at 48 h under different concentrations of *trans*-cinnamic acid.

## Data Availability

The data supporting the conclusions of this article are included with the article and its supplementary material.

## Abbreviations

PAL: Phenylalanine ammonia lyase
4CL: 4-Coumarate-CoA ligase
STS: Stilbene synthase
CoA: Coenzyme A
*Acc*: Acetyl-CoA carboxylase encoding gene
*fabD*: Malonyl-CoA: ACP transacylase encoding gene
*fabB*: β-Ketoacyl-ACP synthase I encoding gene
*fabF*: β-Ketoacyl-ACP synthase II encoding gene
*fabH*: β-Ketoacyl-ACP synthase III encoding gene
