# Supplementary material for: Efficient biosynthesis of pinosylvin from lignin-derived cinnamic acid by metabolic engineering of Escherichia coli
Source: Biotechnol Biofuels Bioprod. 2022 Dec 12;15:136. doi: 10.1186/s13068-022-02236-5 (PMC9743564; doi:10.1186/s13068-022-02236-5)
Supplement: Supplementary file 1 — Additional file 1: Table S1. GenBank IDs and sequences of stilbene synthases used in this study. Table S2. Primers used in this study. Figure S1. Growth curves of E. coli BLS5 cultured in YM9. Figure S2. SDS-PAGE of Ptr4CL4 and PpSTS in BRS4 and BR4S. Figure S3. The OD600 of BR4S at 48 h under different concentrations of trans-cinnamic acid. [file 13068_2022_2236_MOESM1_ESM.docx]

**Supporting information**

**Efficient biosynthesis of pinosylvin from lignin–derived cinnamic acid by Metabolic Engineering of *Escherichia coli***

Yue-Li Hu ^a,b^, Chen Zhang^b^, Li-Hua Zou ^a,b^, Zhao-Juan Zheng ^a,b^, Jia Ouyang ^a,b,^*

^a^ Jiangsu Co-Innovation Centre of Efficient Processing and Utilization of Forest Resources, Nanjing Forestry University, Nanjing 210037, People’s Republic of China;

^b^ College of Chemical Engineering, Nanjing Forestry University, Nanjing 210037, People’s Republic of China;

*Corresponding Author Address: College of Chemical Engineering, Nanjing Forestry University, Nanjing 210037, People’s Republic of China, Tel.: 86-025-85427129, Fax: 86-025-85427587, E-mail: [hgouyj@njfu.edu.cn](mailto:hgouyj@njfu.edu.cn).

Table S1. GenBank ID and sequences of stilbene synthases used in this study.

| Stilbene synthases | Names | GenBank numbers | Source |
| --- | --- | --- | --- |
| PdSTS1 | Pinosylvin synthase | BAA94593.1 | *Pinus densiflora* |
| PdSTS2 | Pinosylvin synthase | BAA89667.1 | *Pinus densiflora* |
| PdSTS3 | Pinosylvin synthase | BAA89668.1 | *Pinus densiflora* |
| PsSTS1 | Pinosylvin synthase | P48407.1 | *Pinus strobus* |
| PsySTS1 | Pinosylvin synthase | Q02323.1 | *Pinus sylvestris* |
| PsySTS2 | Pinosylvin synthase | AAB24341.2 | *Pinus sylvestris* |
| PmSTS1 | Pinosylvin-forming stilbene synthase | ABG91049.1 | *Pinus massoniana* |
| PmSTS2 | Pinosylvin-forming stilbene synthase | ABG91050.1 | *Pinus massoniana* |
| PpSTS | Stilbene synthase | ALN42233.1 | *Pinus pinea* |
| PtSTS | Pinosylvin synthase-like protein | AHK13302.1 | *Pinus thunbergii* |
| Ptr4CL4 | 4-Coumarate-CoA ligase family protein | EEF00197.1 | *Populus trichocarpa* |
| Ptr4CL5 | 4-Coumarate-CoA ligase family protein | EEE79804.2 | *Populus trichocarpa* |
| PsSTS2^Q361R^ | Pinosylvin synthase | - | *Pinus strobus* |
| PsSTS2 | Pinosylvin synthase | P48408.1 | *Pinus strobus* |

Table S2. Primers used in this study.

| Name | Sequence 5’ →3’ | Restriction site |
| --- | --- | --- |
| 4CL5-f | **AAGTATAAGAAGGAGATATA**CATATGATGGATACAATAAC | **NdeⅠ** |
| 4CL5-r | **GCAGCGGTTTCTTTACCAGA**CTCGAGTTACTTTTGCAAAC | **XhoⅠ** |
| 4CL4-P-f | **AACCGCAACCACCATGAGTTAA**CTCGAGTCTGGTAAAGAAAC | **Xho Ⅰ** |
| 4CL4-P-r | **ACGGTGGCAACACTCAT**CATATGTATATCTCCTTCTTATACT | **Nde Ⅰ** |
| STS-f | ATGATGGGCGCTGTTGACTTCGAA |  |
| STS-r | TTATTGCAGCGGAACACTTTTCAG |  |
| RBS-4CL4-f | ATGAGTGTTGCCACCGTGGAACC |  |
| RBS-4CL4-r | TTAACTCATGGTGGTTGCGGTTGC |  |
| RBS-STS-f | ATGGGCGCTGTTGACTTCGAA |  |
| RBS-STS-r | TTATTGCAGCGGAACACTTTTCA |  |
| P-4-f | **AAAAGTGTTCCGCTGCAATAA**CTCGAGTCTGGTAAAGAAACC |  |
| P-4-r | **CGAAGTCAACAGCGCCCAT**CATATGTATATCTCCTTCTTATAC |  |
| plasmids-4CL4-f | **AACCGCAACCACCATGAGTTAA**CGGCCGCATAATCGAAATTAATA |  |
| plasmids-4CL4-r | **TTCCACGGTGGCAACACTCAT**TGTACACTCCTTATACGATTACTTTCTGTTCG |  |
| plasmids-STS-f | **TGAAAAGTGTTCCGCTGCAATAA**CGGCCGCATAATCGAAATTAATAC |  |
| plasmids-STS-r | **TTCGAAGTCAACAGCGCCCAT**TGTACACTCCTTATACGATTACTTTCTGTTCG |  |

Bold sequences were the homologous arm and the underlined sequences were restriction site





Figure S1. The growth curves of *E. coli* BLS5 cultured in YM9 under different concentrations of cerulenin.

Figure S2. SDS-PAGE of Ptr4CL4 and PpSTS in BRS4 and BR4S. Lane 1 represents soluble protein fractions in BRS4. Lane 2 represents insoluble protein fractions in BRS4. Lane 3 represents soluble protein fractions in BR4S. Lane 4 represents insoluble protein fractions in BR4S. The red arrows represent Ptr4CL4 and PpSTS. Ptr4CL4 is about 65.0 kDa. PpSTS is about 45.0 kDa.





Figure S3. The OD_600_ of BR4S at 48 h under different concentrations (50 mg/L, 80 mg/L, 110 mg/L, 130 mg/L, 170 mg/L, 185 mg/L and 200 mg/L) of *trans*-cinnamic acid.
